# Depletion of the mRNA translation initiation inhibitor, programmed cell death protein 4 (PDCD4), impairs L6 myotube formation

**DOI:** 10.14814/phy2.13395

**Published:** 2017-09-12

**Authors:** Naomi Maeda, Abdikarim Abdullahi, Brendan Beatty, Zameer Dhanani, Olasunkanmi A. J. Adegoke

**Affiliations:** ^1^ School of Kinesiology and Health Science and Muscle Health Research Centre York University Toronto Ontario Canada

**Keywords:** Apoptosis, muscle regeneration, myogenesis, PDCD4, skeletal muscle

## Abstract

The mechanistic (mammalian) target of rapamycin complex 1 (mTORC1) signaling is vital for optimal muscle mass and function. Although the significance of mTORC1 in stimulating muscle growth is unequivocal, evidence in support of its role during muscle regeneration is less clear. Here, we showed that the abundance (protein and mRNA) of the mTORC1/S6K1 substrate, programmed cell death protein 4 (PDCD4), is upregulated at the onset of differentiation of L6 and C2C12 cells. The increase in PDCD4 was not associated with any changes in S6K1 activation, but the abundance of beta transducing repeat‐containing protein (*β*‐TrCP), the ubiquitin ligase that targets PDCD4 for degradation, increased. Myoblasts lacking PDCD4 showed impaired myotube formation and had markedly low levels of MHC‐1. Analysis of poly (ADP‐ribose) Polymerase (PARP), caspase 7 and caspase 3 indicated reduced apoptosis in PDCD4‐deficient cells. Our data demonstrate a role for PDCD4 in muscle cell formation and suggest that interventions that target this protein may hold promise for managing conditions associated with impaired myotube formation.

## Introduction

The mammalian/mechanistic target of rapamycin complex 1 (mTORC1) relays growth factors (Dan et al. 2002; Manning et al. 2002; Potter et al. 2002), cellular energy status (Inoki et al. 2003; Kimura et al. 2003), availability of amino acids (especially leucine (Hara et al. 1998; Christie et al. 2002; Beugnet et al. 2003), arginine (Hara et al. 1998; Ban et al. 2004) and glutamine (Nicklin et al. 2009)), and O_2_ economy (Brugarolas et al. 2004; Reiling and Hafen 2004) to discrete cellular processes, including protein synthesis, autophagy, ribosome biogenesis, lipogenesis, and nucleic acid homeostasis (reviewed in (Liko and Hall 2015; Saxton and Sabatini 2017)). Through these processes, activated mTORC1 promotes anabolism. In fact, mTORC1 has been demonstrated to be a critical regulator of muscle mass (Ohanna et al. 2005; Bentzinger et al. 2008; Risson et al. 2009)**.** Given its anabolic characteristics and the fact that it sits at the nexus of cellular substrate availability and synthetic pathways, mTORC1 should be expected to play a role in muscle regeneration. Indeed, inhibition of mTORC1 or muscle‐specific knock‐out of the mTORC1 obligatory substrate‐specifying component, raptor, severely impairs muscle mass and regeneration (Ohanna et al. 2005; Bentzinger et al. 2008; Risson et al. 2009). Also, mice lacking ribosomal protein S6 kinase 1 (S6K1) have impaired muscle development (Ohanna et al. 2005) and inhibition of mTORC1 with rapamycin impairs muscle cell differentiation (Coolican et al. 1997). However, another study contradicts these findings by showing that raptor inhibits differentiation (Ge et al. 2011).

**Figure 1:**
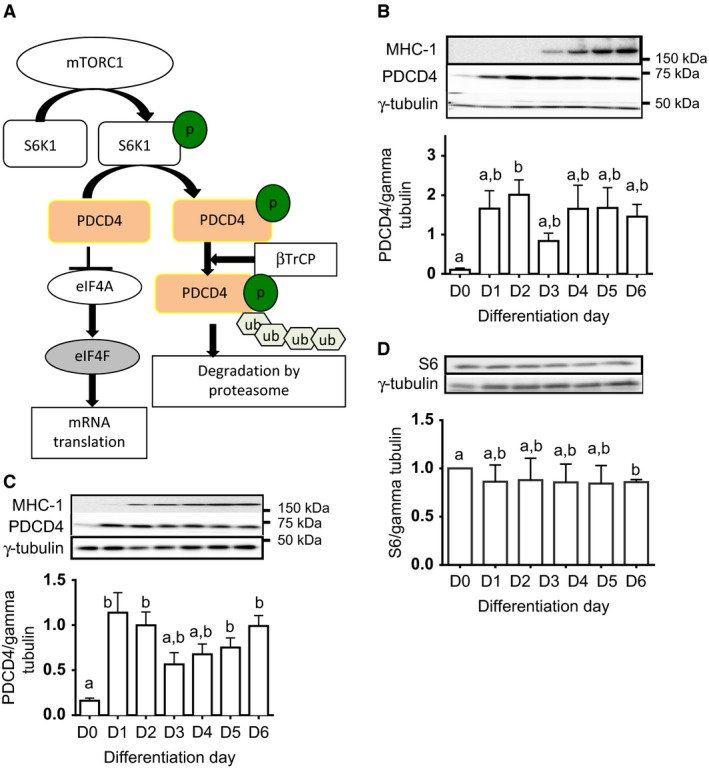
Specific increase in PDCD4 abundance in differentiating muscle cells. A. Simplified scheme of PDCD4 regulation by mTORC1. PDCD4 binds to eukaryotic translation initiation factor 4A (eIF4A). This prevents eIF4A from binding to eIF4E and eIF4G (not shown) to form eIF4F. Inability to form eIF4F impairs mRNA translation initiation. Upon activation, mTORC1 activates S6K1 by phosphorylating its T389 residue. Activated S6K1 then phosphorylates PDCD4 on S67, a modification that targets PDCD4 for polyubiquitination by the ubiquitin protein ligase beta‐transducin repeat containing protein (*β*‐TrCP). Polyubiquitinated PDCD4 is then degraded by the proteasome, a scenario that frees eIF4A for incorporation into eIF4F and therefore favors mRNA translation. p, phosphate group; ub, ubiquitin. L6 (B) and C2C12 (C) were cultured in differentiation medium for 1–6 day. Cell lysates from each day of differentiation were subjected to immunoblotting to detect PDCD4 and MHC‐1. Data are means ± SEM of at least 3 independent experiments. Bars with different letters are significantly different (0.01 <  *P* < 0.05). PDCD4 abundance is significantly higher on D2 (Fig. 1B) and on D1, 2, 5, and 6 (1C) compared to D0. However, the values for D1 to D6 are not significantly different from one another. In D, lysates from L6 cultured in differentiation medium for 1–5 day were probed for ribosomal protein S6. Data are means ± SEM of at least 3 independent experiments. Bars with different letters are significantly different (0.01 < *P* < 0.05). S6 abundance on D5 is significantly lower than on D0, but values for D1 to D5 are not significantly different from one another.

Programmed cell death protein 4 (PDCD4) is one of the substrates of mTORC1/S6K1. In the unphosphorylated state, PDCD4 inhibits mRNA translation via its binding to eukaryotic mRNA translation initiation factor (eIF) 4A and 4G (Yang et al. 2003, 2004). Upon mitogen stimulation, PDCD4 is phosphorylated on S67 by S6K1. This targets PDCD4 for ubiquitination by the ubiquitin protein ligase beta‐transducin repeat‐containing protein (*β*‐TrCP) and subsequent degradation by the proteasome (Fig. 1A, (Dorrello et al. 2006)). The protein kinase AKT too can phosphorylate PDCD4, which causes the protein to be shuttled from the cytoplasm to the nucleus (Palamarchuk et al. 2005). Although the mechanism of action of PDCD4 on mRNA translation initiation is well understood, only very few substrates, including p53 (Wedeken et al. 2011), c‐myb (Singh et al. 2011) and procaspase‐3 (Eto et al. 2012), have been described. In addition to its effect on mRNA translation initiation, PDCD4 can also inhibit translation elongation independent of its binding to eIF4A or eIF4G (Biyanee et al. 2014). Finally, the protein can inhibit transcription of some genes, including those of AP‐1‐dependent transcription (Yang et al. 2001; Zhang et al. 2006).

Much of what is known about PDCD4 is as it relates to oncogenesis. This is because the protein was originally identified as a proapoptotic cell cycle inhibitor and tumor suppressor (Young et al. 2003). Given the function of PDCD4 as a cell cycle inhibitor and a promoter of p21 expression (Goke 2004), it is likely that this protein plays a role in muscle cell differentiation and therefore muscle regeneration. Moreover, PDCD4 is a proapoptotic protein and apoptosis is required for optimal myotube formation (Hochreiter‐Hufford et al. 2013). Here, we hypothesized that PDCD4 level would be upregulated at the initiation of myoblast differentiation and that cells lacking the protein would be impaired in their ability to form myotubes.

## Materials and Methods

### Reagents

Fetal Bovine Serum (FBS), Horse Serum (HS), Lipofectamine RNAiMax, OptiMEM, and antibiotic/antimycotic (Ab‐Am) reagents were purchased from Life Technologies (Burlington, Ontario, Canada). Control and PDCD4 siRNA oligonucleotides were purchased from Sigma‐Aldrich (St. Louis, MO). Phosphatase and protease inhibitor cocktails were purchased from Sigma‐Aldrich. *α*‐ Modification of Eagle's Medium (AMEM) and Phosphate Buffered Saline (PBS) were obtained from Wisent (St‐Bruno, Quebec, Canada).

### Antibodies

Antibodies to PDCD4, phosphorylated (ph)‐S6K1 (T389), S6, ph‐AKT (S473), caspase 3, caspase 7, poly (ADP‐ribose) Polymerase (PARP), and GAPDH were purchased from Cell Signaling Technology (Danvers, MA). Monoclonal antibodies to MHC‐1 and myogenin were developed by Drs. Fischman D.A. and Wright W.E., respectively, and were obtained from the Developmental Studies Hybridoma Bank, created by the NICHD of the NIH (University of Iowa, Iowa City, Iowa). Antibodies to *γ*‐tubulin were purchased from Sigma‐Aldrich.

### Cell culture and treatments

L6 rat skeletal muscle myoblasts were obtained from the American Type Culture Collection. Cells were cultured in 6‐well plates (150–250 × 10^3^ cells/well) or in 10‐cm plates (600 × 10^3^ cells/plate). They were propagated at 37°C and 5% CO_2_ in humidified atmosphere in proliferation medium composed of AMEM supplemented with 10% FBS and 1% Ab‐Am until they reached at least ~90% confluency. Cells were then either harvested (day 0) or shifted into differentiation medium (DM) (AMEM supplemented with 2% HS and 1% Ab‐Am). They were harvested day 1–5 during differentiation. Differentiation medium was replaced with fresh medium every other day. To ascertain that our observations were not limited to L6 cells, some of the experiments were also carried out in C2C12. In such experiments, C2C12 were cultured as above but in DMEM growth medium (DMEM, 10% FBS, 1% Ab‐Am). For differentiation, cells were cultured in DMEM‐based differentiation medium (DMEM, 2% HS and 1% Ab‐Am).

### Cell fractionation

Cells cultured in 10‐cm plates cells were trypsinized with 1 mL trypsin/plate. Five mL ice‐cold PBS was added to stop the trypsin reaction. Cells were centrifuged (620*g*, 5 min) and pellets resuspended in 1 mL of PBS. They were then centrifuged (430*g*, 3 min at 4°C). After discarding the supernatant, pellets were resuspended in 500 *μ*L of Buffer 1 (10 mmol/L Tris, pH 7.4, 10 mmol/L NaCl, 3 mmol/L MgCl_2_, 0.5% NP‐40, 10 *μ*L/mL protease inhibitor cocktail and 10 *μ*L/mL phosphatase inhibitor cocktail). The samples were put on ice and vortexed at low speed for 15 sec every minute for 5 min. The resulting lysate was centrifuged (430*g*, 3 min at 4°C). After centrifugation, supernatants were collected as cytosol. Pellets were washed 2X with 200 *μ*L Buffer 1. They were then resuspended in 150 *μ*L of Buffer 2 (50 mmol/L Tris, pH 7.4, 5 mmol/L MgCl_2_, 0.1 mmol/L EDTA, 1 mmol/L dithiothreitol, 40% (wt/vol) glycerol, 10 *μ*L/mL protease inhibitor cocktail, and 10 *μ*L/mL phosphatase inhibitor cocktail). This was the nuclear fraction.

### RNA interference

L6 myoblasts (250 × 10^3^ cells) were seeded in 6‐well plates. To deplete the cells of PDCD4, cells were transfected with control or PDCD4‐targetting RNAi oligonucleotides to 30 nmol/L final concentration, using Lipofectamine RNAiMax and following manufacturer's protocol (Life Technologies). We used two different PDCD4 siRNA oligonucleotides: PDCD4 #1 sense (GUCUUCUACUAUUACCAUA [dT] [dT]), PDCD4 #1 antisense (5′UAUGGUAAUAGUAGAAGAC [dT] [dT]), PDCD4 #2 sense (CUACUAUUACCAUAGACCA [dT] [dT]), and PDCD4 #2 antisense (UGGUCUAUGGUAAUAGUAG [dT] [dT]). After 48 h of incubation with the transfection mix, cells were harvested in lysis buffer (25 mmol/L Tris, pH 7.5, 1 mmol/L EDTA, 2% sodium dodecyl sulfate (SDS), protease inhibitor cocktail (10 *μ*L/mL), phosphatase inhibitor cocktail (10 *μ*L/mL), and DTT (1 mmol/L)), or switched to DM. Differentiating cells were harvested D1 through D5.

### Quantitative reverse transcriptase PCR

RNA was extracted with TRIzol Plus RNA Purification Kit (Life Technologies), following the manufacturer's protocol. Reverse transcription (RT) was done with iScript Advanced cDNA Synthesis Kit for RT‐qPCR (Bio‐Rad Laboratories Ltd Life Science Group). The resulting cDNA was diluted 1.5 times with autoclaved deionized water. We used primers shown in Table 1 to do quantitative RT‐PCR, which was conducted with SsoAdvanced Universal SYBR Green Supermix (Bio‐Rad Laboratories), using the Bio‐Rad CFX‐96 Touch machine.

**Table 1:** List of primers used for quantitative RT‐PCR analyses

Gene	Forward primer (5′‐3′)	Reverse primer (5′‐3′)
PDCD4	ATGAGACTGTGGTTCTGCCC	TCCCTTAACATCTCCGCGAC
Myogenin	CCCAGTGAATGCAACTCCCA	CGAGCAAATGATCTCCTGGGT
Myosin heavy chain‐1	GAGTCCCAGGTCAACAAGCTG	GTGCCTCTCTTCGGTCATTC
HPRT	CTTCCTCCTCAGACCGCTTTT	ATCACTAATCACGACGCTGG

### Immunoblotting

For experiments involving nuclear‐cytosolic fractionation, 30 *μ*L (nuclear fraction) or 25 *μ*L (cytosolic fraction or whole cell lysate) samples were loaded per well of 15% SDS‐polyacrylamide gel electrophoresis (PAGE). For RNAi experiments, equal amounts of protein (25 *μ*g) were loaded per well of 12.5% gel and subjected to electrophoresis. Proteins were then transferred onto polyvinylidene difluoride (PVDF) membrane. Membranes were incubated overnight at 4°C with desired primary antibodies, followed by incubation with horseradish peroxidase‐conjugated goat anti‐rabbit (GAR) (for PDCD4, GAPDH, histone H2A, caspase 3 and 7, and PARP)) or goat anti‐mouse (GAM) (for MHC‐1, myogenin, troponin and *γ*–tubulin) secondary antibodies for 1 to 3 h at room temperature. Further processing, image collection, and analyses were as described (Kakade et al. 2014).

### Statistical analyses

Data are presented as means ± SEM. Data were analyzed, using one‐way analysis of variance (ANOVA), except in Fig. 4B–D and 5A–C where two‐way ANOVA was used**.** Tukey's post hoc tests were used to identify means that were significantly different from one another. Because of the variability in the datasets for S6, ph‐S6K1, and ph‐AKT1, those data were normalized to D0 values and analyzed, using column statistics. The level of significance was set at *P* < 0.05. Statistical analyses were done using GraphPAD (Version 6, GraphPAD Software, La Jolla, CA).

## Results

### PDCD4 expression increases during L6 and C2C12 myoblast differentiation

In L6 cells, PDCD4 expression significantly increased at the onset of differentiation, especially on D2 (Fig. 1B, *P* < 0.05). MHC‐1 expression was used as a marker of myotube formation (Fig. 1B and C). To ascertain that the effects observed were not limited to a single cell line, we also studied C2C12 and showed that as in L6, PDCD4 level increased at the onset of differentiation (Fig. 1C, compare D1 and D2 to D0). The increase in PDCD4 at the onset of differentiation was not associated with a global increase in the abundance of mTORC1/S6K1 substrates, as the levels of ribosomal protein S6 (Fig. 1D) and of eukaryotic initiation factor 4E‐binding protein 1 (4E‐BP1, data not shown) were not affected. The increase in PDCD4 abundance during differentiation did not significantly affect the fraction of the protein that was associated with eIF4A (data not shown).

### Nuclear and cytosolic expression of PDCD4 in differentiating myoblasts

**Figure 2:**
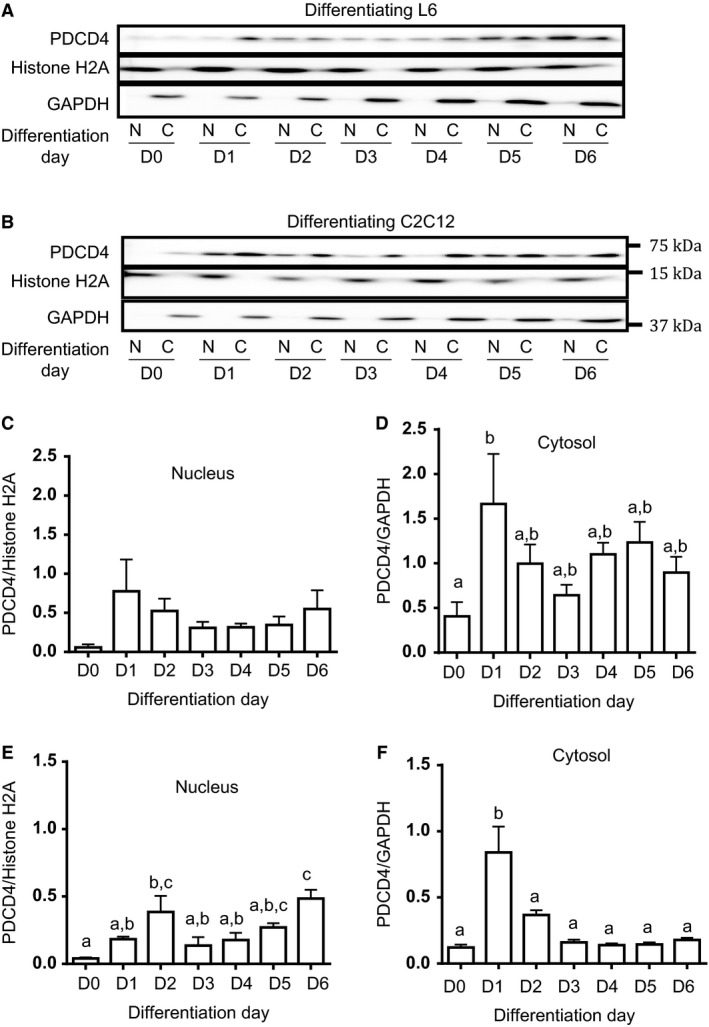
Nuclear and cytoplasmic expression of PDCD4 in differentiating muscle cells. L6 (A, C, D) and C2C12 (B, E, F) were cultured in differentiation medium as described in Figure 1. On D0 and on each day of differentiation, cells were fractionated into nuclear and cytoplasmic components. PDCD4 in each fraction was detected by immunoblotting. Histone H2A and GAPDH were used, respectively, as nuclear and cytoplasmic markers. Data are means ± SEM of at least 3 independent experiments. Bars with different letters differ significantly (0.001 < *P* < 0.05). In Figure 2D, day 1 value is significantly different from D0 but not from values for any of the other days. In Figure 2E, D2 and D6 are significantly different from D0, and D6 is different from any other day, except D2 and D5. In Figure 2F, D1 is significantly different from any other day.

PDCD4 can shuttle between the nucleus and the cytoplasm (Bohm et al. 2003). During differentiation, nuclear PDCD4 tended to increase (L6 cells, Fig. 2A, C) and was significantly elevated on D2 in differentiating C2C12 (Fig. 2B, E). Cytosolic expression of PDCD4 increased on D1 in both L6 (Fig. 2A, D) and C2C12 (Fig. 2B, F), a pattern similar to that seen in whole cell lysate (Fig. 1).

**Figure 3:**
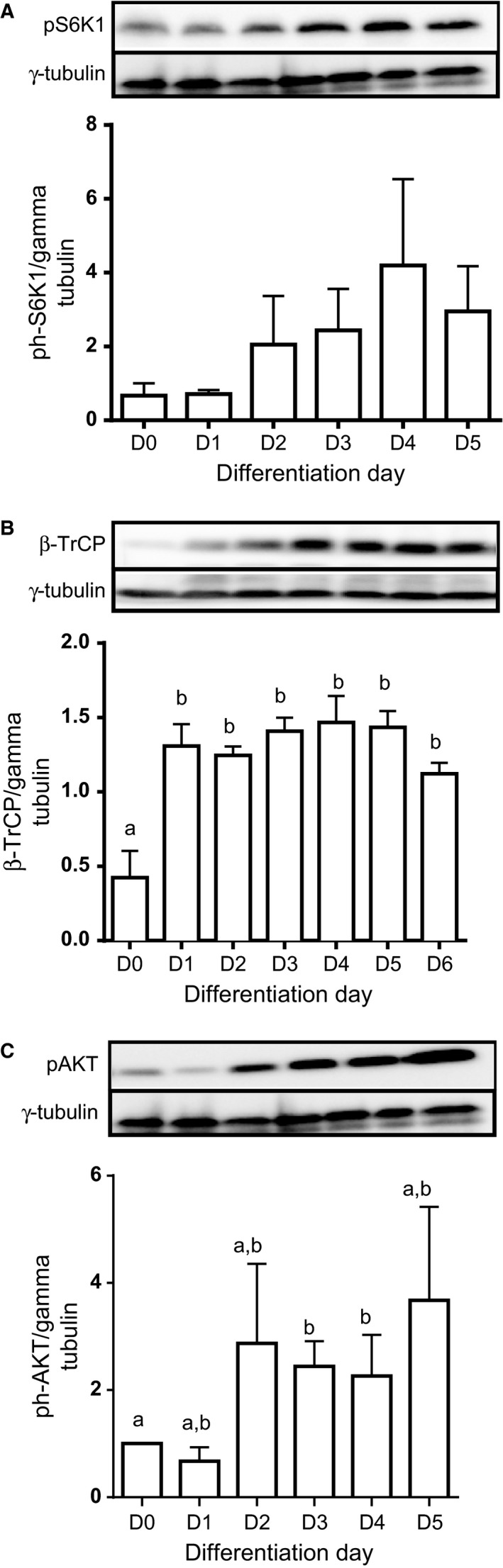
L6 myoblasts were cultured in differentiating medium as described in Figure 1. Cell lysates from each day of differentiation were analyzed for phosphorylated S6K1 (A), *β*‐TrCP (B) and phosphorylated AKT (C). Data are means ± SEM of at least 3 independent experiments. Bars with different letters differ significantly (0.001 < *P* < 0.05). In Figure 3B, D0 is significantly lower than D1‐D6. In Figure 3C, D3, and D4 are significantly different from D0.

To examine the mechanism behind the regulation of PDCD4 in differentiating muscle cells, we examined the phosphorylation state of S6K1, the kinase that phosphorylates PDCD4 and targets it for ubiquitin‐dependent proteolysis (Dorrello et al. 2006). There was no significant effect of differentiation on S6K1 T389 phosphorylation, although there was a trend toward increased phosphorylation of the protein as differentiation proceeded (Fig. 3A). Interestingly, the abundance of *β*‐TrCP, the protein ligase that catalyzes phosphorylation‐dependent ubiquitination of PDCD4 (Dorrello et al. 2006), was low in proliferating cells but increased dramatically at the onset of differentiation (Fig. 3B). AKT too phosphorylates PDCD4 and this is thought to regulate its nuclear‐cytoplasmic shuttle (Palamarchuk et al. 2005). As with S6K1, AKT S473 phosphorylation was not significantly modified during differentiation, except on D3 and 4 when its levels were significantly higher than on D0 (Fig. 3C).

### Myotube formation is impaired in myoblasts depleted of PDCD4

**Figure 4:**
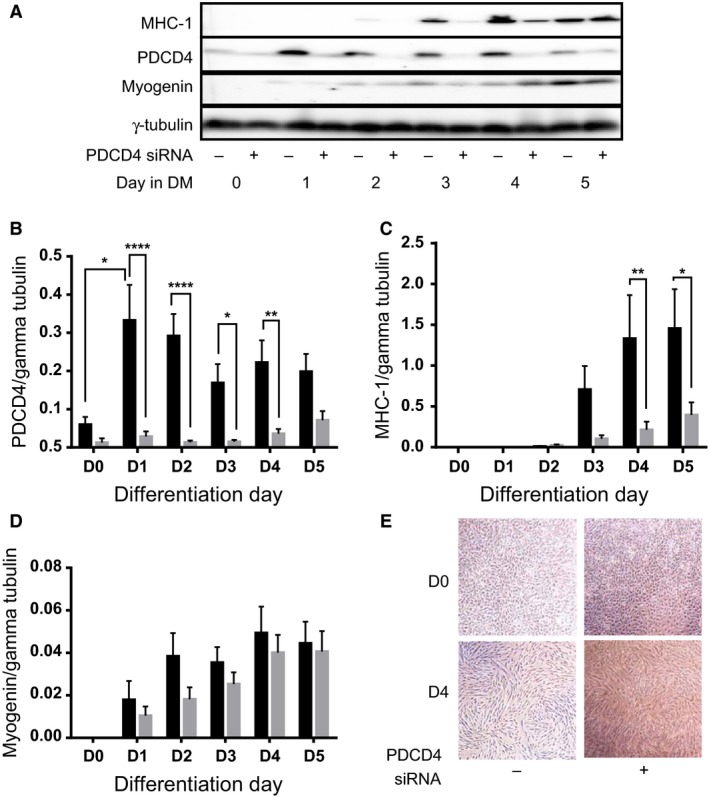
L6 myoblasts were transfected with control (‐, dark bars in B–D), or siRNA oligonucleotides designed against PDCD4 (+; gray bars in B–D). Forty eight h later, cells were shifted into differentiation medium (D0). Lysates from cells harvested from D0 to D5 were analyzed for PDCD4 (A, B), MHC‐1 (A, **C**), and myogenin (A, D). In E, images captured from cells observed under a light microscope are shown. Data are means ± SEM of at least 3 independent experiments. *P < 0.05, **P < 0.01, ****P < 0.0001.

To examine the role of PDCD4 during myotube formation, we employed RNAi to knock down the protein (Fig. 4A and B) and then subjected the myoblasts to the differentiation protocol. Unlike in control cells, in which differentiation was apparent, cells depleted of PDCD4 showed impaired myotube formation as can be seen from light microscope images (Fig. 4E) and expression of myosin heavy chain‐1 (MHC‐1, Fig. 4A, C). Myogenin expression tended to be lower at every time point in cells depleted of PDCD4 (Fig. 4A, D).

**Figure 5:**
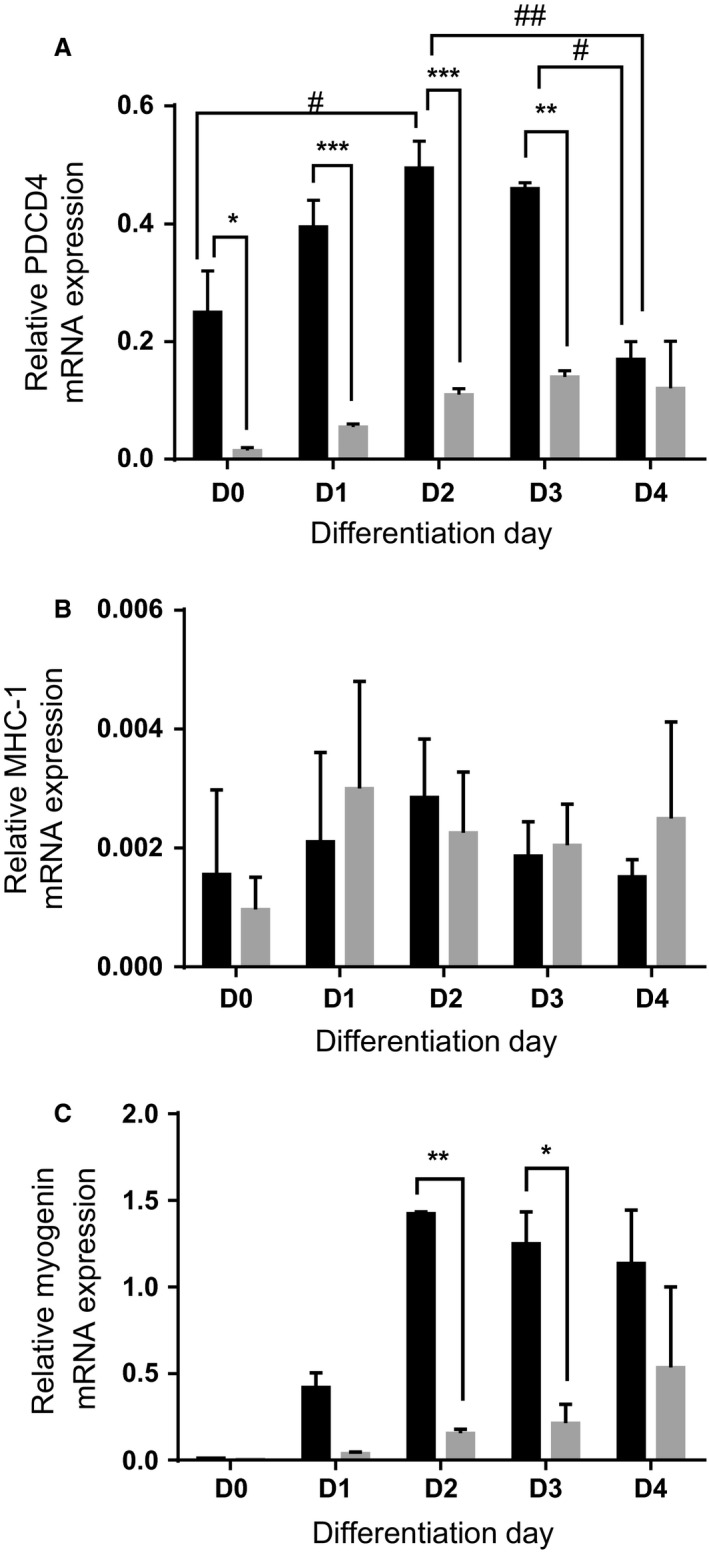
Cells were treated as described in Figure 4. RNA isolated from cells were subjected to quantitative RT‐PCR to detect mRNA for PDCD4 (A), MHC‐1 (B), and Myogenin (C). mRNA data are expressed relative to hypoxanthine phosphoribosyltransferase (HPRT) mRNA. Data are means ± SEM of at least 4 independent experiments. *and # *P* < 0.05, ** and ## *P* < 0.01, ***P < 0.001.

In cells treated with control siRNA, abundance of PDCD4 mRNA level increased, especially between d 0 and d 2 (Fig. 5A) in parallel with the increase in PDCD4 protein abundance seen at the onset of differentiation (Fig. 1 and 4A and B). There were no changes in MHC‐1 mRNA (Fig. 5B) but on d 2 and 3, myogenin mRNA was significantly lower in PDCD4‐depleted cells (Fig. 5C).

### Impaired apoptosis in cells lacking PDCD4

**Figure 6:**
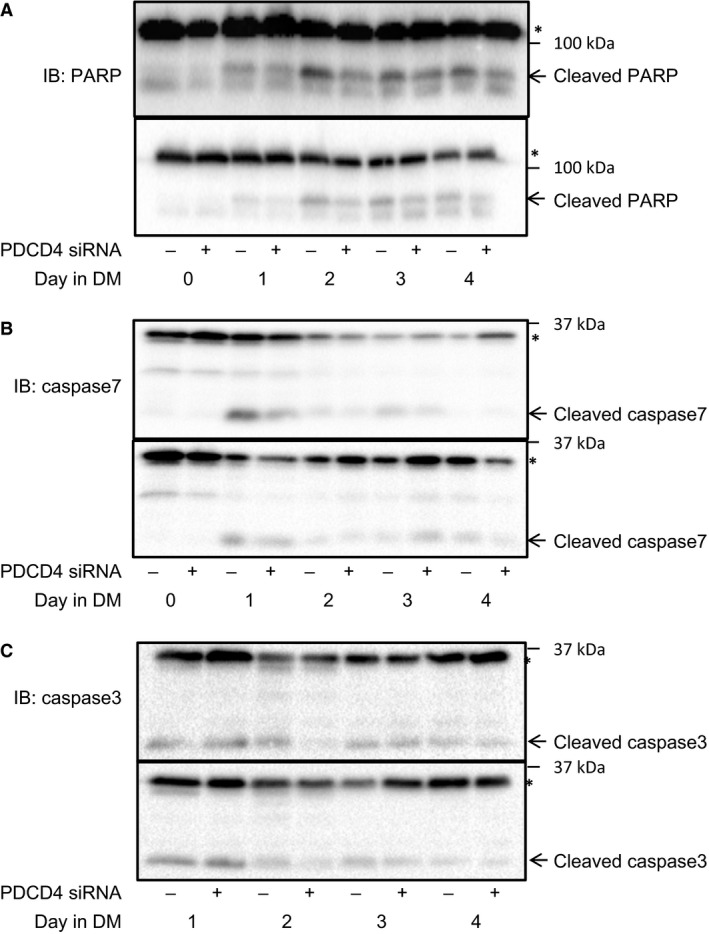
Cells were treated as described in Figure 4. Lysates were probed with anti‐PARP (A), caspase 7 (B), and caspase 3 (C) antibodies. Two blots from three independent experiments are shown. In C, note that data from D1 to D4 of differentiation are shown. In all cases, * indicates the uncleaved protein.

Because PDCD4 is a proapoptotic protein and apoptosis is required for muscle cell differentiation (Hochreiter‐Hufford et al. 2013), we examined the levels of cleaved and uncleaved PARP (Crawford and Wells 2011), caspase 7 and caspase 3 (Oliver et al. 1998). Compared to control cells, there was decreased amount of cleaved PARP in PDCD4‐depeleted cells, especially on D2 of differentiation (Fig. 6A). There were also decreased levels of cleaved caspases 7 and 3 on D1 and/or D2 of differentiation (Fig. 6B and C). These data are consistent with the roles of PDCD4 as a proapoptotic protein and suggest that impairment in myotube formation in cells lacking PDCD4 is related to impaired apoptosis.

## Discussion

Although evidence for the requirement of mTORC1 in the regulation of muscle mass is incontrovertible (Ohanna et al. 2005; Bentzinger et al. 2008; Risson et al. 2009), data on the requirement for mTORC1/S6K1 during muscle cell differentiation and regeneration have been inconclusive. Here, we showed that the abundance of the mRNA translation initiation inhibitor PDCD4, a substrate of mTORC1/S6K1, was specifically upregulated at the onset of differentiation. This occurred in parallel with an increase in *β*‐TrCP level, although without detectable changes in S6K1 phosphorylation. Critically, myoblasts depleted of PDCD4 were impaired in their ability to differentiate and this occurred in parallel with altered levels of markers of apoptosis.

Given its significance in regulating muscle mass (Adegoke et al. 2012) and that raptor knockout mice have impaired muscle formation (Bentzinger et al. 2008), it should be expected that mTORC1 would regulate muscle cell differentiation, a step that is vital for muscle formation and during muscle repair. However, data on the significance of mTORC1 during myotubes formation are inconclusive. For example, myoblasts treated with rapamycin have impaired IGF1‐induced differentiation of L6 myoblasts (Coolican et al. 1997) and kinase activity of mTOR is required for differentiation of C2C12 cells (Shu et al. 2002). However, other studies indicate that raptor knockout enhances differentiation of C2C12 myoblasts (Ge et al. 2011) and that cells lacking S6K1 have normal myotube formation (Ohanna et al. 2005). Here, we showed that not only is PDCD4 upregulated at both the protein and mRNA levels during the onset of differentiation, cells depleted of the protein have impaired myotube formation.

The increase in PDCD4 abundance during differentiation was not simply a result of migration of the protein from the nucleus to the cytoplasm (Fig. 2). The most well characterized regulation of PDCD4 abundance is initiated by mTORC1/S6K1‐dependent phosphorylation, which then targets the protein for ubiquitination and degradation (Dorrello et al. 2006). We made several attempts, using antibodies from two different suppliers, to characterize PDCD4 phosphorylation during differentiation but were unsuccessful in getting reliable signals. We did not observe a significant change in S6K1 phosphorylation during differentiation, something that would have been expected given its role in catalyzing the phosphorylation of PDCD4. However, we note that the level of *β*‐TrCP, the ubiquitin protein ligase that catalyzes ubiquitination of PDCD4 (Dorrello et al. 2006), increased from d 0 to d 1 and remained elevated through d 5. This suggests that elevated PDCD4 (seen on d 1 and 2) might trigger mechanisms that will increase the degradation of the protein, albeit in a phosphorylation‐independent manner. Indeed, cullin 3‐ring finger protein ubiquitin ligase (CRL3^IBTK^) can target PDCD4 for degradation, independent of a requirement for phosphorylation (Pisano et al. 2015). Whether *β*‐TrCP too can target PDCD4 for degradation in a phosphorylation‐independent manner remains to be demonstrated.

The fact that mRNA level of PDCD4 (Fig. 5A), in addition to its protein level, changes suggests that altered transcription of PDCD4 contributes, at least in part, to its dynamic regulation during differentiation. The change in PDCD4 mRNA might be related to diminished levels of miRNAs, such as miR‐21 (Allgayer 2010), miR‐182 (Wang et al. 2013) and miR‐320a (Wang et al. 2016), that negatively target 3′ UTR of PDCD4 mRNA. miR‐21 is expressed in muscle and, contrary to what one might expect, is proapoptotic (He et al. 2014). Whether this and the other miRs that target PDCD4 regulate muscle cell differentiation remains to be discovered.

That PDCD4 depletion led to a marked reduction in myoblast differentiation suggests a critical role for this protein during muscle development and regeneration. But by what mechanism/s does PDCD4 regulate differentiation? The profound effect of its knock down on myogenin mRNA, in the absence of little or no effect on MHC‐1 mRNA, suggests both transcriptional and nontranscriptional effects. The most well understood mechanism of action of PDCD4 relates to its negative effect on mRNA translation initiation (Allgayer 2010). Therefore, an obvious mechanism of effect of PDCD4 would be via regulation of translation of MHC. However, an opposite outcome should have been seen if MHC and myogenin mRNA are targets of PDCD4. This suggests that PDCD4 might target factors that are negative regulators of myofibrillar protein abundance.

Because it is a proapoptotic protein and apoptosis is required for myoblast differentiation (Hochreiter‐Hufford et al. 2013), another possible mechanism of action of PDCD4 is via regulation of programmed cell death. In this regard, we note that impaired differentiation observed in cells depleted of PDCD4 occurred in parallel with impaired apoptosis, as measured by fractional cleavage of PARP, caspases 3 and 7. Additional studies are needed to identify the specific mechanisms by which PDCD4 regulates apoptosis during the differentiation of muscle cells.

A limitation of this work is that it is an in vitro study. In vivo studies, for example, with muscle damage‐regeneration model, and with primary cell lines derived from cachectic patients, will be useful to extend our findings. Our study provides data that will be useful in future research in this area.

In conclusion, we demonstrated that PDCD4 is required for myotube formation. This suggests that interventions that stabilize PDCD4 might have therapeutic potentials against muscle damage and wasting.

## Conflict of Interest

The authors declare no conflict of interest.
